# Shame Mediates the Relationship Between Pain Invalidation and Depression

**DOI:** 10.3389/fpsyg.2021.743584

**Published:** 2021-12-03

**Authors:** Brandon L. Boring, Kaitlyn T. Walsh, Namrata Nanavaty, Vani A. Mathur

**Affiliations:** ^1^Department of Psychological and Brain Sciences, Texas A&M University, College Station, TX, United States; ^2^Texas A&M Institute for Neuroscience, College Station, TX, United States

**Keywords:** mental health, self-worth, social support, pain, discounting, pain invalidation

## Abstract

The experience of pain is subjective, yet many people have their pain invalidated or not believed. Pain invalidation is associated with poor mental health, including depression and lower well-being. Qualitative investigations of invalidating experiences identify themes of depression, but also social withdrawal, self-criticism, and lower self-worth, all of which are core components of shame. Despite this, no studies have quantitatively assessed the interrelationship between pain invalidation, shame, and depression. To explore this relationship, participants recounted the frequency of experienced pain invalidation from family, friends, and medical professionals, as well as their feelings of internalized shame and depressive symptoms. As shame has been shown to be a precursor for depression, we further explored the role of shame as a mediator between pain invalidation and depressive symptoms. All sources of pain invalidation were positively associated with shame and depressive symptoms, and shame fully mediated the relationship between each source of pain invalidation and depression. Relative to other sources, pain invalidation from family was most closely tied to shame and depression. Overall, findings indicate that one mechanism by which pain invalidation may facilitate depression is *via* the experience of shame. Future research may explore shame as a potential upstream precursor to depression in the context of pain. Findings provide more insight into the harmful influence of pain invalidation on mental health and highlight the impact of interpersonal treatment on the experiences of people in pain.

## Introduction

Pain is an often invisible affliction with neither an obvious outward expression nor a unifying underlying pathology that together with its subjective nature makes it difficult to be supported and/or understood by social circles and healthcare providers alike (Kool et al., 2009, 2010). Lay people, nurses, and doctors frequently think that others are experiencing less severe pain than they report (Guru and Dubinsky, 2000; Marquié et al., 2003; Panda et al., 2006; Mathur et al., 2014; Ng et al., 2019), or sometimes do not believe they are in pain at all (Kool et al., 2009), thus discounting their pain and invalidating their experience. Pain invalidation can involve discounting and/or lack of understanding: Discounting specifically involves disbelieving, dismissing, or punishing a person in pain because of their expression of pain, whereas lack of understanding may come from people who acknowledge an individual’s pain but do not consider it serious, consequential, or requiring support (Kool et al., 2010; Nicola et al., 2019). Importantly, pain invalidation is associated with detrimental effects on mental health and subjective well-being, including greater negative affect, pain disability, pain severity, and most notably, depression (Vangronsveld and Linton, 2012; Kool et al., 2013; Crow et al., 2014; Edlund et al., 2017; Wernicke et al., 2017; Molzof et al., 2020). In addition, qualitative research indicates that experiences of pain invalidation evoke experiences of stigmatization – a societal judgment that enforces shame – and other constructs that are closely tied to shame, such as lower self-worth and social withdrawal (Asbring and Närvänen, 2002; Jones et al., 2004; Drossman et al., 2009; Oehmke et al., 2009; Nicola et al., 2019, 2021). Further support for the relationship between pain invalidation, shame, and depression comes from qualitative research indicating people often feel shame and shame-related constructs, such as self-consciousness, due to the experience of pain itself (regardless of exposure to invalidation) and that illness invalidation broadly is associated with depression (Osborn and Smith, 1998; Smith and Osborn, 2007; Sehlo et al., 2016; Boring et al., 2021). However, no studies have examined shame and depression together directly in relation to pain invalidation.

Shame is a self-conscious emotion characterized by global feelings of inadequacy and low self-worth (Tangney and Dearing, 2003). Shame has been linked to myriad negative psychological constructs, such as depression, anxiety, stress, PTSD, paranoia, and non-suicidal self-injury (Andrews et al., 2000; Robinaugh and McNally, 2010; Pinto-Gouveia and Matos, 2011; Johnson et al., 2014; Schoenleber et al., 2014; Bannister et al., 2018). Shame is also related to pain experiences in general; shame proneness has been found to be higher in those with chronic pain relative to those without (Turner-Cobb et al., 2015), and shame is a common theme described in qualitative reports examining pain experiences (Asbring and Närvänen, 2002; Turner-Cobb et al., 2015; Nicola et al., 2019). Those suffering from chronic pain have reported that the shame they feel is more unbearable than the pain itself (Smith and Osborn, 2007). Scholars have described pain as “an assault on the self” because it leads people to question themselves and their experiences, similar to the ruminative and critical self-views held by shame-prone individuals (Smith and Osborn, 2007; Tangney et al., 2007; Milia et al., 2021). Additionally, people living with chronic lower back pain have described “comparing the self with others” and “withdrawing from others,” both of which are behaviors exhibited by those feeling shame (Osborn and Smith, 1998; Tangney et al., 2007). As such, the shame that often is described in relation to the experience of pain itself may be further compounded by pain invalidation.

Ultimately, the power of invalidation lies in its attack on subjective experiences that cannot be objectively observed by others. Experiencing invalidation of any kind is an interpersonal harm that negatively impacts health (Tanaka et al., 2011; Westphal et al., 2016). Invalidation in other domains (e.g., emotional invalidation) is associated with poorer mental health, including shame proneness, depression, anxiety, bipolar disorder, and suicidal behaviors (Johnson et al., 2002; Krause et al., 2003; Fruzzetti et al., 2005; Yap et al., 2008; Crow et al., 2014; Mahtani et al., 2018, 2019; Naismith et al., 2019). Furthermore, the relationship between shame and other interpersonal harms, such as abuse and neglect, has consistently been documented (Feiring and Taska, 2005; Robinaugh and McNally, 2010; Pinto-Gouveia and Matos, 2011; La Bash and Papa, 2014). Frequent exposure to emotional invalidation, abuse, and neglect can lead to the internalization of these events as representative of self, harmfully mirroring the unfounded thoughts of others that one is “globally defective” (Pinto-Gouveia and Matos, 2011; Matos and Pinto-Gouveia, 2014). It is thus similarly possible that consistent exposure to pain invalidation may begin to cause the individual to question or discount not just their pain, but their core feelings and experiences as well, and to hide their pain to meet the expectations of others, gradually developing into internalized shame (Asbring and Närvänen, 2002; Nicola et al., 2019). When these invalidating and shame provoking events become central to one’s sense of self, people are then increasingly likely to experience more intense symptoms of depression (Robinaugh and McNally, 2010). Indeed, shame has been recognized as a precursor for the development of depression and may be a primary driver of the established link between pain invalidation and depression (Birk, 2013; Mills et al., 2015).

One limitation of previous studies on pain invalidation and depression is that they have focused on patients with chronic pain, where confounding and mutually reinforcing relationships between these experiences may limit interpretation. Examination of acute pain invalidation might allow for exploration of potential preclinical predictors of depressive symptoms. Further, as acute pain is a primary reason for seeking medical attention (i.e., nearly 50% of all emergency healthcare visits (Chang et al., 2014)), opportunity for invalidation of acute pain in clinical contexts is high. Considering that depression increases the risk of transition from acute pain to chronic pain (Magni et al., 1994; Currie and Wang, 2005; Apkarian et al., 2013), identifying pathways that contribute to depression in acute pain contexts is clinically relevant for both the physical and mental health of the person in pain. However, invalidation of acute pain has received limited attention. Extant studies have observed invalidation in relation to controlled pain applied in the laboratory rather than to naturally occurring pain (e.g., muscle soreness and headache) and have assessed aspects of the pain experience, such as tolerance and intensity or broad psychological constructs (e.g., positive/negative affect) with mixed results (Linton et al., 2012; Moore et al., 2013; Birnie et al., 2017; D’Agostini et al., 2020; Pester et al., 2020). Furthermore, pain invalidation within these studies was often manipulated or influenced by the experimenters, which may not convey the same connotations and magnitude of invalidation that could organically arise during typical social interaction. Importantly, no previous studies have examined the interrelationship between invalidation of naturally occurring acute pain, shame, and depression.

In the current study, we bridge the gaps above and examine the interrelationship between pain invalidation, shame, and depression. Using self-report surveys, we examined bivariate correlations between frequency of experiences with pain invalidation from three separate domains (family, friends, and medical professionals) and shame and depressive symptoms. To control for potential confounding and mutually reinforcing relationships in the context of chronic pain and to explore potential preclinical predictors, we explored these relationships among young adults without chronic pain. We hypothesized that people who have had their pain invalidated from any source more frequently would report greater shame and depression. Furthermore, as shame is often a precursor for the development of depression, we hypothesized that shame would mediate the relationship between frequency of invalidation experiences and depression (Birk, 2013; Mills et al., 2015).

## Materials and Methods

### Participants

Participants (*n*=478; *M*_age_=18.48, ± 0.82years old; 328 women, 139 men, 1 identified as “other” (unspecified gender identity), and 10 chose not to disclose gender; and 270 White, 111 Hispanic/Latinx, 53 Asian, 21 multiracial, 9 Black, 2 American Indian/Alaskan Natives, 1 identified as “other” and specified “Hispanic/White,” and 11 chose not to disclose racialized group identity) were recruited from a student research pool from September to November, 2020. Inclusion criteria were being at least 18years of age and free from current chronic pain. Thirty-five participants reported current naturally occurring acute pain (e.g., non-chronic muscle soreness and headache), and 21 were currently taking pain medication. This study was approved by the Texas A&M Institutional Review Board; all participants provided electronic informed consent to agree to participate.

### Procedure

This study was part of a larger project assessing the role of shame in pain experiences. The measures described here were presented first. Other measures (e.g., assessment of social and environmental factors) were included to support exploratory secondary analyses. Interested participants completed the study online and were compensated with course credit. The survey was created and accessed within Qualtrics (Provo, UT).^1^ The survey took approximately 30min to complete.

### Materials

#### Pain Invalidation

Pain invalidation was assessed using the Illness Invalidation Inventory (3^*^I; Kool et al., 2010). Similar to previous studies, the survey was modified slightly to specifically assess invalidation of pain (Molzof et al., 2020). Furthermore, due to the undergraduate population, the sources of Work Environment and Social Services were not included from the original survey, and the domain of Friends was added to tap into more likely and relevant domains encountered by this group. Participants recounted the frequency with which they experienced invalidation about their pain over the previous year from three separate sources – Family (*α*=0.728), Friends (*α*=0.691), and Medical Professionals (*α*=0.658) – in the way described by each item (Kool et al., 2010). Specifically, participants were prompted with “We are interested in how others react to people experiencing pain. Each of the sections below refers to different people in your life. We would like you to rate how often during the past year each person or category of people reacted toward you in the way described. After each statement, select a number between 1 (never) and 5 (very often) to indicate how often they reacted toward you that way. The questionnaire has 3 sections, and you will rate the same reactions a number of times, but referring to different people. If a particular section does not apply to you, you may skip that part of the questionnaire and go on to the next section. Remember, rate the items with respect to how others reacted toward you as a person when experiencing pain.” Eight items were asked for each source (e.g., *My family makes me feel like an exaggerator*) using a scale of 1 (never) to 5 (very often). The eight items were averaged within each domain, such that higher total scores represent more frequent experiences of pain invalidation.

#### Shame

Shame was assessed using the 24 shame items (e.g., *I feel insecure about others’ opinions of me*) of the internalized shame scale (*α*=0.929) using a response scale from 0 (never) to 4 (almost always; Cook, 1988). Total scores were summed such that higher scores indicate greater internalized shame.

#### Depression

Depression was assessed using the 20-item (e.g., *I was bothered by things that do not usually bother me*) Centers for Epidemiological Studies – Depression scale (CES-D; *α*=0.926; (Radloff, 1977). Participants responded using a 0 (rarely or none of the time) to 3 (most or all of the time) scale, and total scores were summed such that higher scores indicate greater depression.

### Analysis Plan

Analyses were conducted using SPSS (version 25; IBM Corp, Armonk, NY). First, we conducted bivariate correlations to examine the relationship between pain invalidation from each domain with shame and depression. We then probed shame as a mediator between pain invalidation from each separate source and depression using Hayes’ PROCESS macro. Confidence intervals were evaluated based on bias-corrected bootstrapping with 5,000 permutations. To control for Type I error increased by conducting three-related mediation models, we used a Bonferroni adjusted *α* of 0.017 to determine statistical significance. Although not the focus of the current research, we also examined these relationships within-group (i.e., gender and racialized group identity) as an initial test of generalizability.

## Results

Descriptive statistics for the predictor and outcome variables are reported in Table 1. Prevalence of pain invalidation from all sources was very high in this sample: 99.4% of participants reported some (3^*^I score>1) experience of invalidation from family members, 98.9% from friends, and 95.5% from their doctors. Within this sample, the full range of possible responses across invalidation experiences and shame scores was observed. Nearly, a full range of scores for symptoms of depression was seen (max CES-D score=57), with 59.3% of participants meeting or exceeding the recently suggested cutoff score of 20 indicating a risk for clinical depression (Vilagut et al., 2016).

**Table 1:** Descriptive statistics and bivariate correlations for primary variables of interest.

Descriptive Statistics					Bivariate Correlations (*r*)			
Variable	Min	Max	Mean	SD	Invalidation: Friends	Invalidation: Medical	Shame	Depression
Invalidation: Family	1.00	4.63	2.37	0.66	0.349^*^	0.411^*^	0.443^*^	0.347^*^
Invalidation: Friends	1.00	4.50	2.25	0.59	–	0.406^*^	0.264^*^	0.250^*^
Invalidation: Medical	1.00	4.13	1.85	0.53		–	0.254^*^	0.239^*^
Shame	24.00	120.00	61.45	24.23			–	0.781^*^
Depression	4.00	57.00	24.75	12.33				–

* *p*<0.001.

**Figure 1:**
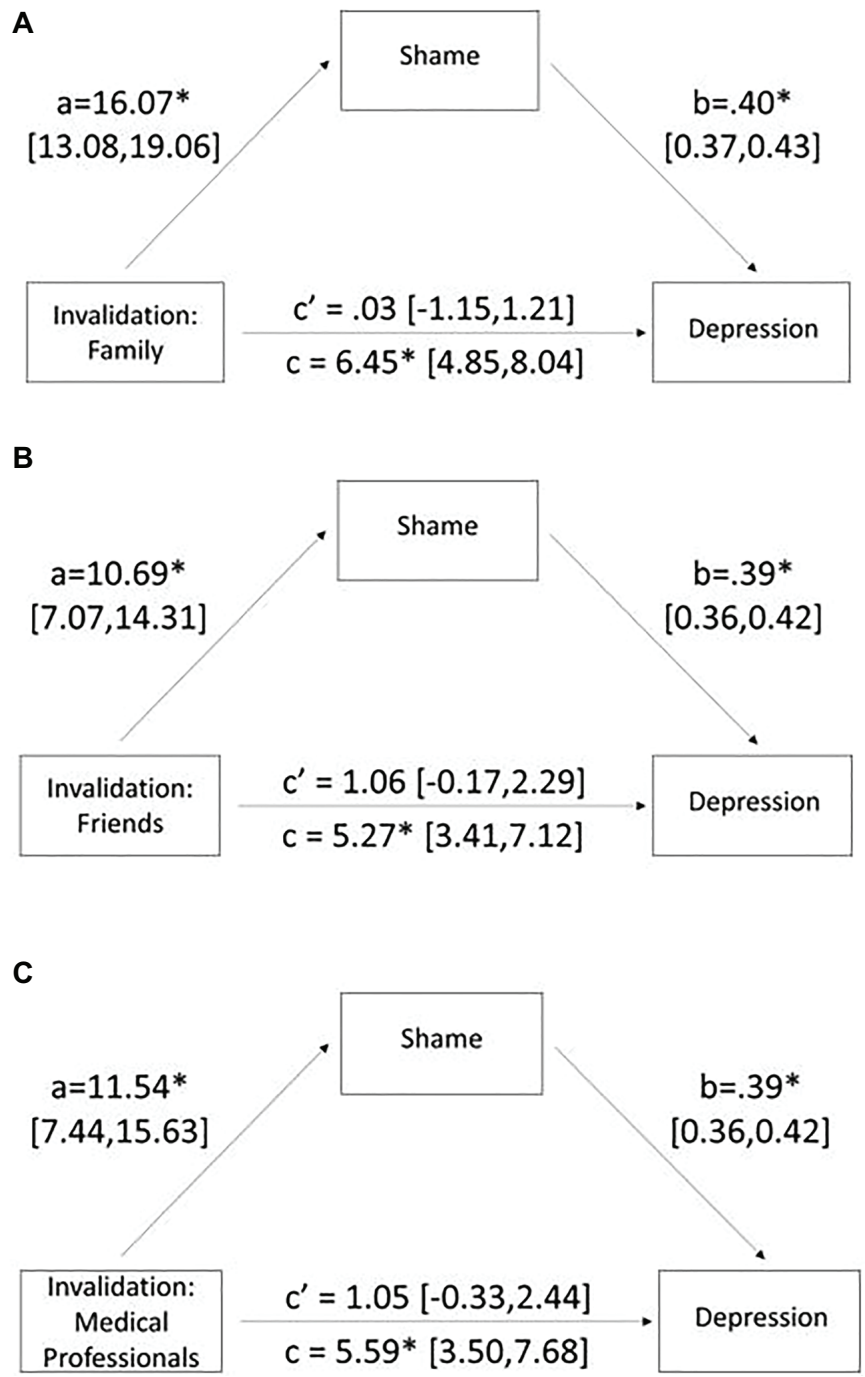
Mediation analysis with pain invalidation as the predictor, shame as the mediator, and depression as the outcome variable for **(A)** pain invalidation from family, **(B)** pain invalidation from friends, and **(C)** pain invalidation from medical professionals. Numbers represent unstandardized betas, and brackets indicate 95% confidence intervals. ^*^p<0.001.

Greater frequency of pain invalidation from any source was associated with greater shame and depressive symptoms (0.250≤*r*≤0.443; Table 1). Mediation analyses revealed that pain invalidation from family (*B*=6.45, 95% CI [4.85, 8.04], *p*<0.001), friends (*B*=5.27, 95% CI [3.41, 7.12], *p*<0.001), and medical professionals (*B*=5.59, 95% CI [3.50, 7.68], *p*<0.001) had a significant positive total effect on depressive symptoms. The confidence intervals of the indirect effects of pain invalidation from family (*B*=6.42, 95% CI [5.11, 7.79]), friends (*B*=4.20, 95% CI [2.73, 5.66]), and medical professionals (*B*=4.53, 95% CI [2.73, 6.36], *p*<0.001) on depression through shame also did not include zero. However, this relationship was driven by shame which fully mediated the relationship between invalidation and depression, such that the direct effect of invalidation on depressive symptoms was substantially reduced and was no longer significant for family (*B*=0.03, 95% CI [−1.15, 1.21], *p*=0.96), friends (*B*=1.06, 95% CI [−0.17, 2.29], *p*=0.09), or medical professionals (*B*=1.05, 95% CI [−0.33, 2.44], *p*=0.13). Pain invalidation from each domain strongly predicted increased feelings of shame, which in turn predicted greater symptoms of depression (Figure 1). Although not the focus of the current research, we assessed if these results held within certain groups. Results were consistent for men and women separately, except for men/doctor invalidation, with the overall model not significant but trending in the same direction. The results were also consistent for White and Latinx (the most represented minoritized population) participants across all invalidation domains.

## Discussion

Pain invalidation is the undermining of the subjective experience of pain by people external to the experience and has previously been linked to depression. Here, we provide evidence that one mechanism by which this interpersonal experience influences depression is through shame. Shame fully mediated the relationship between frequency of pain invalidation – whether from one’s family, friends, or medical professionals – and depression symptoms. Shame is ultimately an interpersonal emotion that involves gauging one’s self in relation to others’ expectations, whether that be behavioral or emotional in nature. When other people invalidate a person’s pain, they may communicate that the person in pain is not worthy of empathy or support. This in turn may cause the person suffering from pain to question their own subjective state and their value as a human, creating feelings of shame, and inadequacy. Doubting one’s self-worth while still experiencing pain that others do not believe may culminate in depressive states (Mirels et al., 2002; McGregor et al., 2008). This is particularly concerning considering the facilitating role of depression in the transition of acute pain to chronic pain (Magni et al., 1994; Currie and Wang, 2005; Apkarian et al., 2013). To the extent that invalidating acute pain experiences contribute to depression through shame, it may also compound the mutually reinforced depression-pain relationship and increase the risk prolonged trajectories of pain. Although frequency of invalidation experiences was intercorrelated across domains, and all were associated with shame and depression, experiences were distinct and invalidation from family was most strongly correlated with shame. While future studies are needed to support interpretation of this finding, it is possible that those who experience pain invalidation in their home environments may also be subjugated to other forms of invalidation (e.g., emotional) and neglect that also evoke feelings of shame, compounding experiences that could contribute to depression (Mahtani et al., 2018, 2019; Naismith et al., 2019). This may clarify prior research that has demonstrated emotional invalidation from family is associated with depression as well as negative affect, physiological stress reactions (e.g., increased heart rate and skin conductance), reduced social engagement, and maladaptive behavioral responses to distress (Krause et al., 2003; Mountford et al., 2007; Yap et al., 2008; Shenk and Fruzzetti, 2011; Crow et al., 2014; Greville-Harris et al., 2016).

The current study also provides further insight into the social nature of pain. While pain is subjective and felt only by the person experiencing it, the expression and communication of pain exist to enable the person suffering from pain to receive aid (Craig, 2015). Social support (or lack thereof as observed in pain invalidation) is known to contribute to the overall experience of pain, but the specific psychosocial mechanisms through which this occurs are still being uncovered (López-Martínez et al., 2008; Woods et al., 2019). Here, we show that the self-conscious emotion of shame is a potential link between social interactions – specifically pain invalidation – and personal health outcomes in the pain experience. This is partially supported by prior research showing that shame mediates the association between abuse and somatization, as well as between neglect and somatization, suggesting that shame is a key mechanism in facilitating painful health outcomes following forms of interpersonal/social harms (Kealy et al., 2018). As such, the relationship between experiences of pain invalidation, another interpersonal harm, and increased pain severity and disability may also be driven by shame (Molzof et al., 2020).

This is also the first study to our knowledge to examine invalidation of naturally occurring (i.e., not induced in a laboratory) acute pain. The present result that acute pain invalidation is commonly experienced even by healthy young adults points to the prevalence of pain invalidation as a sociocultural norm affecting clinical and non-clinical samples of all ages. Furthermore, we show that experiencing invalidation of acute pain may be harmful to mental health, similar to effects previously demonstrated in chronic pain. As acute pain is highly prevalent in clinical settings, invalidation of acute pain and its correlates of mental health warrant further clinical and empirical attention. Future research may also examine the potential role of invalidation-related increases in depression through shame and the development of chronic pain.

There were limitations to this study that should constrain interpretation. First, the sample was composed of young adults and may therefore not be representative of all those who have experienced pain invalidation. However, the frequency with which the participants indicated they had experienced invalidation suggests that pain invalidation is pervasive and relevant – even among young people without chronic pain. With that said, the relationship between invalidation, shame, and depression should be explored among those suffering from chronic pain to further understand the clinical implications of the current findings. In the current study, we also did not collect information about the type or source of pain that was invalidated by others. Although participants were not suffering from chronic pain at the time of their participation, we are not able to rule out the possibility that some may have experienced chronic pain within the last year nor are we able to distinguish between the range of acute pains that could have received varying levels of invalidation. Such investigations may be the focus of future research. Additionally, we assessed general internalized shame and not shame specifically in response to the invalidating experiences. However, the experiences may be intertwined regardless of assessment of the direct source of feelings of shame, as other findings have linked invalidating experiences with general shame (Mahtani et al., 2018, 2019; Naismith et al., 2019). Future studies may examine pain invalidation-specific shame to determine if this enhances prediction of depression or more fully mediates the relationship between invalidation and depression. Finally, while the percentage of participants at risk for clinical depression may seemingly be higher than what has typically been found in college students (~46%), it must be noted that these data were collected during the COVID-19 pandemic which had been shown to increase symptoms of depression among this group (Vilagut et al., 2016; Acharya et al., 2018; Wang et al., 2020); future studies should attempt to replicate this outside of the presence of a global stressor.

Pain is frequently underestimated or dismissed by others, and the experience of having one’s subjective pain experience invalidated confers additional harm and suffering (Guru and Dubinsky, 2000; Marquié et al., 2003; Panda et al., 2006). Validation of pain from providers, family members, and social circles alike (and promotion of such validation through clinical and community intervention) has the capacity to impact psychological repercussions associated with the experience of pain. Listening to the pain reported by patients and validating their subjective pain experience may foster interpersonal trust between patients and providers and improve pain outcomes. Furthermore, broader cultural understanding about the subjective nature of pain and the importance of social support for those experiencing pain may protect against the mutually reinforcing depression-pain cycle. Ultimately, invalidation of one’s internal pain experience may foment shame through self-doubt, self-criticism, and social withdrawal that drives the established link between invalidation and depression. Addressing feelings of shame and validating subjective pain experiences may decrease the burden of depression in the context of pain.

## Data Availability Statement

The raw data supporting the conclusions of this article will be made available by the authors, without undue reservation.

## Ethics Statement

The studies involving human participants were reviewed and approved by Texas A&M University Institutional Review Board. The patients/participants provided their written informed consent to participate in this study.

## Author Contributions

BB, KW, and VM contributed to study conception and design. BB and KW collected data. BB conducted data analysis and wrote the first draft of the manuscript. All authors (BB, KW, NN, VM) edited the manuscript, have read the final draft, and have approved submission.

## Funding

NN was supported by a NSF Graduate Research Fellowship.

## Conflict of Interest

The authors declare that the research was conducted in the absence of any commercial or financial relationships that could be construed as a potential conflict of interest.

## Publisher’s Note

All claims expressed in this article are solely those of the authors and do not necessarily represent those of their affiliated organizations, or those of the publisher, the editors and the reviewers. Any product that may be evaluated in this article, or claim that may be made by its manufacturer, is not guaranteed or endorsed by the publisher.

## Footnotes

^1^ Qualtrics.com
